# The Synergistic Role of *Sargassum horneri* Fucoidan and *Lactobacillus plantarum*: Microbiome and Gut Barrier Restoration in Zebrafish Colitis

**DOI:** 10.3390/md23100372

**Published:** 2025-09-25

**Authors:** N. M. Liyanage, D. P. Nagahawatta, H. H. A. C. K. Jayawardhana, Jun-Geon Je, Li Yiqiao, Fengqi Yang, Young-Sang Kim, Kyung Yuk Ko, You-Jin Jeon

**Affiliations:** 1 Department of Marine Life Sciences, Jeju National University, Jeju 690-756, Republic of Korea; 2 Department of Medicine, Faculty of Medicine and Dentistry, University of Alberta, Edmonton, AB T6G 2B7, Canada

**Keywords:** DSS-induced colitis, *S. horneri*, fucoidan, synbiotics, microbial dysbiosis, tight junction proteins, alpha diversity

## Abstract

Ulcerative colitis (UC) is a chronic inflammatory bowel disease marked by disruption of the intestinal barrier and gut microbiota imbalance, leading to significant impairment in patient quality of life. This study investigated the therapeutic efficacy of a synbiotic formulation composed of purified fucoidan from bloom-forming *Sargassum horneri* and the probiotic *Lactobacillus plantarum* in a dextran sulfate sodium (DSS)-induced zebrafish model of UC. Polysaccharides from *S. horneri* were extracted using Celluclast-assisted extraction and fractionated via DEAE anion-exchange chromatography, resulting in six fucoidan fractions. The sixth fraction (SH-F), with a molecular weight of 254 kDa, showed the highest fucose, sulfate contents, and demonstrated the highest effect on promoting *L. plantarum* growth. Structural analysis revealed that SH-F contained α-L-Fucp-(1→3), α-L-Fucp-(1→4), β-D-Galp-(1→2,3,4), α-L-Fucp-(1→3,4), and terminal α-L-Fucp residues where Fuc_1_(SO_3_)_1_, Gal1Fuc_1_(SO_3_)_1_, and Fuc_2_(SO_3_)_2_ were the most common glycans. Synbiotic administration significantly attenuated DSS-induced colonic shrinkage, inhibited pro-inflammatory cytokines (IL-6, TNF-ɑ, and IL-1β), restored tight junction proteins (ZO-1, occludin), and downregulated the iNOS, COX2, and NF-κB signaling pathway in adult zebrafish. 16S rRNA gene sequencing revealed restoration of gut microbial diversity and increased abundance of beneficial bacterial taxa to improve DSS-induced UC. These findings highlight the potential synergistic effects of SH-F and *L. plantarum* as a combinatorial strategy to regulate gut inflammation and enhance epithelial barrier function, potentially offering new insights and therapeutic opportunities for UC intervention.

## 1. Introduction

Inflammatory bowel disease (IBD) is a chronic, non-specific inflammatory condition affecting the gastrointestinal tract, encompassing ulcerative colitis (UC) and Crohn’s disease (CD), posing a significant global health challenge [1]. UC is an idiopathic intestinal disorder marked by recurring cycles of inflammation and remission in the colonic mucosa. This condition significantly impairs patients’ quality of life and imposes substantial costs on healthcare systems [2]. Although the underlying causes of chronic inflammation are not fully understood, it is reported that complex interactions among genetic, environmental, and immunological factors play a role [3]. A key regulator in the inflammatory cascades associated with UC is the transcription factor nuclear kappa B (NF-κB). These play a crucial role in orchestrating inflammatory responses by activating numerous pro-inflammatory genes, thereby driving inflammation, oxidative stress, and immune reactions that contribute to UC progression [4]. Moreover, changes in gut microbiome, both in terms of type and quantity, are considered a crucial factor in the progression of UC. This is particularly evident when comparing UC patients to healthy individuals [5]. Chronic inflammatory conditions in the gut result in increased intestinal permeability, disruption of the structure of the intestinal mucosal barrier, disturbance of gut microbiota homeostasis, and depletion of beneficial intestinal synbiotic bacteria. These combined factors lead to the amplification of the immune response, ultimately contributing to disease recurrence and exacerbation [6].

Conventional treatments for UC, including surgery and medications (salicylates, immunosuppressants), come with drawbacks such as postoperative complications, side effects, and substantial costs. This underscores the pressing need for novel and natural UC treatment approaches. Probiotics such as lactic acid bacteria, *Bifidobacterium* spp., which help in maintaining gut homeostasis, have the potential to prevent various intestinal diseases [7]. Previous studies have demonstrated that treatment with probiotic bacteria induces interleukin-10 (IL-10)-producing macrophages in inflamed mucosa, repairs tight junctional protein damage, and promotes the regeneration of intestinal lymphatic vessels [8]. *Lactobacillus plantarum* is considered a primary probiotic that regulates gut microbiota balance, enhances the adhesion of beneficial bacteria to the intestinal mucosa, inhibits the adhesion of pathogenic bacteria, and suppresses inflammatory reactions [9]. Several animal and clinical trials have reported that *L. plantarum* reduces the chronic inflammation in the gut in patients with UC and relieves the experimental colitis induced by dextran sulfate sodium (DSS) [10,11]. Moreover, it is proven that *L. plantarum* can restore the damaged mucosal barrier function and enhance the intestinal immune system [12].

Synbiotics, which are the combination of specific strains of probiotic bacteria and selected prebiotics, generally show a greater efficacy than either component alone [2]. Synbiotics composed of β-glucan and a complex probiotic formulation significantly modulated inflammatory cell infiltration and reduced levels of inflammatory markers, including interleukin-6 (IL-6) and tumor necrosis factor-alpha (TNF-α), in C57BL/6 mice. These synbiotics exhibited stronger and broader inhibitory effects on colonic inflammation compared to β-glucan or probiotics administered individually [13].

Fucoidan, a sulfated polysaccharide, has emerged as a promising synbiotic component for the treatment of UC due to its prebiotic and anti-inflammatory activities. However, commercial fucoidan usually contains multiple peaks of molecular weights, therefore causing their activities to vary greatly. Homogeneous bioactive fucoidans were prepared and purified, demonstrating significant potential for health-promoting applications. Fucoidans are complex polysaccharides derived from brown seaweeds, composed primarily of L-fucose along with other monosaccharides and sulfated ester groups [14]. Fucoidan isolated from brown seaweed *Sargassum horneri* has been recognized as a good prebiotic material due to its potential to improve the growth and function of probiotic bacteria as well as its anti-inflammatory potential. The *S. horneri,* which is the source of the fucoidan of our study, is identified as a dominant seaweed linked with golden tide along the coastal areas of China, Japan, and South Korea [15]. While *S. horneri* is recognized as a major contributor to golden tides in Korea, there is a notable gap in the in-depth examination of the biological activities associated with this *S. horneri* fucoidan. In our previous study, we reported that treatment of sulfated polysaccharide from *S. horneri* improved the probiotic bacterial growth and reduced the pathogenic bacterial infection in zebrafish, proving itself as a promising candidate for enhancing gut health [16]. Moreover, the synbiotic treatment of *S. horneri* fucoidan and *L. plantarum* resulted in a synergistic anti-inflammatory effect in DSS-induced in vitro cell and in vivo zebrafish larvae [17]. Given the capacity of fucoidan to support probiotic growth and function, they may synergistically interact with probiotics to improve UC by strengthening barrier function, modulating inflammatory cytokines, regulating gut microbiota, and promoting immune system development.

Therefore, the current study was undertaken to investigate the synbiotic effect of *Lactobacillus plantarum* and bloom-forming *S. horneri* fucoidan in a DSS-induced UC zebrafish model. We hypothesized that co-treatment would address UC by downregulating inflammation in the intestine, protecting the integrity of the intestinal barrier, and maintaining the balance of the gut microbiota.

## 2. Results

### 2.1. S. horneri Fucoidan Increases the Growth of L. plantarum

**Figure 1:**
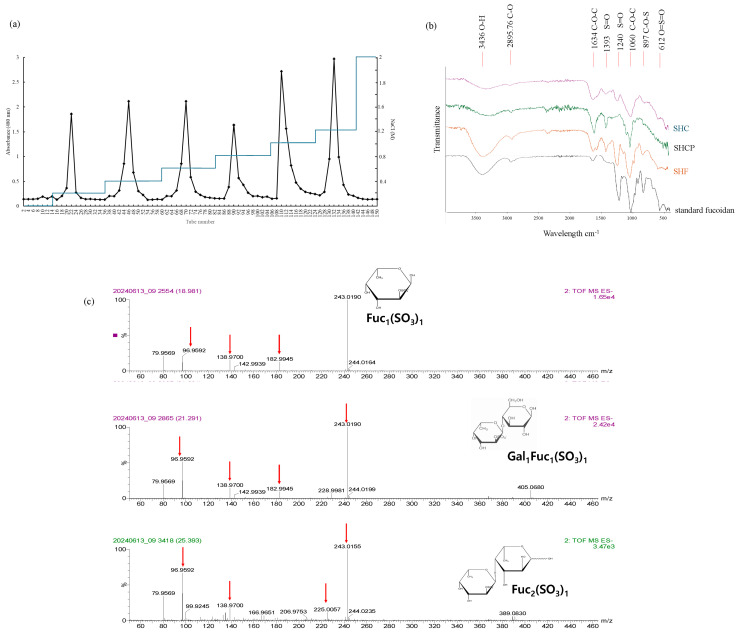
Characterization of SH-F. (**a**) Purification of *S. horneri* fucoidan, (**b**) FTIR chromatographs, and (**c**) Ms/MS spectra of common glycans in SH-F.

Sulfate possesses an anion charge; therefore, anion-exchange chromatography is the most suitable for separating into high yield, and we have used DEAE-cellulose for this purpose. DEAE anion-exchange chromatography of SHCP yielded six distinct fucoidan fractions as SH-F1-SH-F6 (Figure 1a). The yield and purity of each fraction are given in Supplementary Table S2. The yield was calculated using the weight of the raw material, and the amount obtained accordingly was smaller than that of the crude sulfated polysaccharide content.

Treatment of *S. horneri* fucoidan fractions in the culture medium of *L. plantarum* resulted in higher growth of the probiotic bacteria (Supplementary Figure S2). The highest growth was observed in the sixth fucoidan fraction-treated group (680.00 ± 18.85%). Therefore, the sixth fucoidan fraction (SH-F) was selected for future experiments.

### 2.2. Purified S. horneri Fucoidan Fraction by Anion-Exchange Chromatography

Chemical composition analysis revealed that SHCP was composed of 50.17 ± 0.44% total polysaccharides and 15.00 ± 0.24% sulfate. SH-F contained sulfate content of 32.21 ± 0.12% and polysaccharide content of 62.09 ± 0.47% (Supplementary Table S3). According to monosaccharide analysis, SH-F contained higher amounts of fucose (49.43 ± 0.17%) and galactose (20.05 ± 0.31%) compared to the other monosaccharides. It is proposed that MALS be used to determine the average size and molecular weight of the absolute molar mass and the molecular weights of the polymer. SH-F was eluted with PL aquagel-OH MIXED-H and detected in the RI detectors. SH-F was detected with a molecular weight of 2.54 × 10^6^ g/mol.

### 2.3. Fourier Transform Infrared Spectroscopy (FTIR) Analysis

FTIR was performed to compare and analyze the functional groups of SHC, SHCP, and SH-F with commercial fucoidan. As shown in Figure 1b, SHC, SHCP, and SH-F showed distinct peaks at 2981 cm^−1^, suggesting the presence of a C-O bond in a methyl group, a distinctive functional group found in fucose. Additionally, the peaks at 1393 and 1241 cm^−1^ are associated with sulfate groups (S=O). A peak at 1060 cm^−1^ shows the C-O bond in the aromatic ring structure. The peak at 897 cm^−1^ is responsible for the C-O-S bond. The intensity peak at 1240 cm^−1^, responsible for S=O was higher in SH-F, indicating higher sulfate ester content. The peak at 897 cm^−1^ was associated with the C-O-S bond.

### 2.4. UPLC-QTOF-MS Analysis

The results of UPLC-Q-TOF MS analysis for SH-F are shown in Figure 1c. As for the peak setting condition, integrated processing inherent in MassLynx v4.1 SCN888 was used, and data processing was performed by setting it to peak-to-peak 1000. The produced fractions via acid hydrolysis are used to identify three main glycans. The selected MS chromatogram peaks confirmed the precursor ion mass values, and the main glycans in SH-F are shown in Figure 1c. Fuc_1_(SO_3_)_1_ (243.02 *m*/*z*,C_6_H_11_O_8_S^−^), Fuc_2_(SO_3_)_1_ (389.08 *m*/*z*,C_12_H_20_O_11_S^−^), and Gal1Fuc_1_(SO_3_)_1_ (405.07 *m*/*z*, C_12_H_22_O_13_S^−^) are the main glycans in fractions, and this result was analyzed by checking the fragment for each. The glycan composition of SH-F was assessed using exact effective mass (*m*/*z*), which was produced through the UNIFI software.

### 2.5. Linkage Pattern Analysis

Based on standard data and retention time in the Complex Carbohydrate Research Center (CCRC) spectral database, the ratios of the partially methylated alditol acetates of SH-F were identified, as listed in Table 1. The fucose-based sugar residues (1,3-linked Fucp, 1,4-linked Fucp, and 1,3,4-linked Fucp with proportions of 0.44, 0.16, and 0.16, respectively) were highly enriched in SH-F. Moreover, 1,6-linked Gal and 1,4,6-linked Gal, with proportions of 0.11 and 0.13, respectively, were present in SH-F. This suggests that 1,3-linked Fucp and 1,4-linked Fucp are the main components of the backbone of SH-F. Furthermore, only 1,3-linked Fucp and 1,4-linked Fucp were detected, suggesting that polysubstituted Galp was also the main component of the backbone of SH-F to build the branch chain.

### 2.6. Synbiotic Treatment of L. plantarum and SH-F on Body Weight and Mortality of Zebrafish

**Figure 2:**
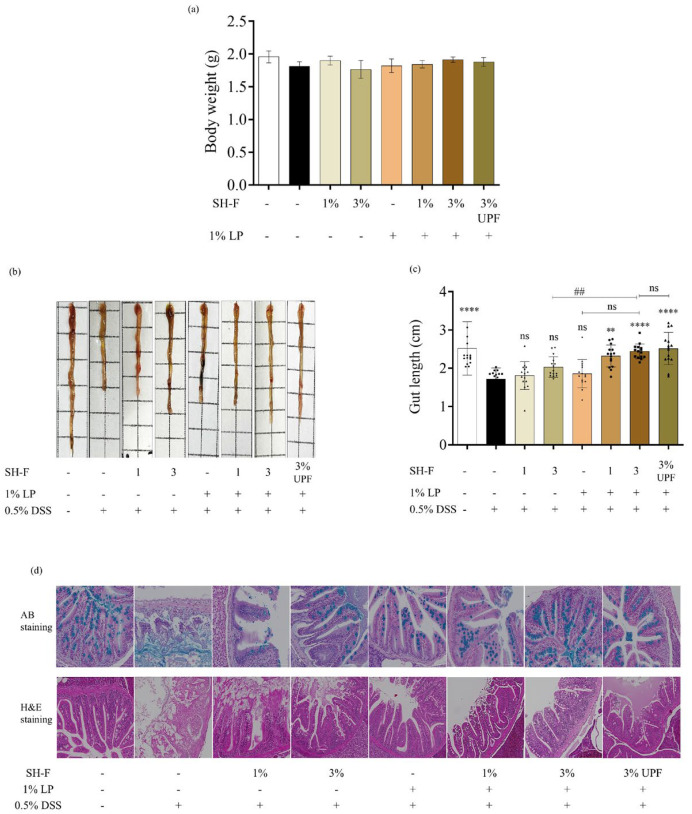
Effects of synbiotic treatment of *L. plantarum* and SH-F on body weight, intestinal length, and histopathological morphology in 0.5% DSS-induced adult zebrafish. (**a**) Body weight at the end of synbiotic treatment period, (**b**) representative intestines from the treatment groups, (**c**) intestinal length, and (**d**) H&E and Alcian blue staining of colon tissue sections. The results are represented as means ± SD. Values are significantly different from the control group (0(+)) at ** *p* < 0.01, and **** *p* < 0.0001, and from 3% SH-F+LP group at ## *p* < 0.01. (SH-F: *S. horneri* fucoidan, LP: *L. plantarum*, UPF: *U. pinnatifida* fucoidan).

No mortality of zebrafish was observed during the experiment period. The experimental diet was fed to zebrafish for 14 days and weight gain was recorded before 0.5% DSS exposure. The effects of diet on growth performance are shown in Figure 2a. No significant difference in body weight was observed among groups. The *Undaria Pinnatifida* fucoidan (UPF) was used as a positive control in this study due to its proven anti-inflammatory and prebiotic activity [18].

### 2.7. Synbiotic Treatment of SH-F and L. plantarum Reduced Morphological Changes and Injuries in the Intestine

Morphological changes in the intestine, including the shortened length and tissue damage, occur in the intestinal inflammatory model of zebrafish. According to the obtained results, intestinal length was significantly decreased in 0.5% DSS-exposed fish (Figure 2b,c). The 0.5% DSS-treated group induced a significant reduction in gut length (1.71 ± 0.3 cm) compared to the untreated group (2.55 ± 0.71 cm), reflecting the impact of inflammation on the intestinal morphology. However, synbiotic treatment of SH-F and *L. plantarum* showed a distinct positive effect on intestinal length compared to their individual treatments.

Histological examination of the colon tissues was performed using H&E and Alcian Blue staining (Figure 2d). Alcian blue staining of the intestinal section of zebrafish indicated the presence of mucin in goblet cells. Abundant healthy goblet cells were observed in the intestinal tissues of the untreated group, whereas a significant decrease in goblet cells was noted in the 0.5% DSS-treated group. In contrast, synbiotic treatment of SH-F and *L. plantarum* resulted in a considerable increase in the number of goblet cells present in the intestine. Moreover, H&E staining of the 0.5% DSS-treated group showed notable histopathological alterations, including crypt deterioration and submucosal edema, compared to the untreated group. However, treatment of SH-F, *L. plantarum,* and their combined treatment effectively mitigated the 0.5% DSS-induced intestinal damage and inflammation in the zebrafish intestine, preserving the colon tissue morphology.

### 2.8. SH-F and L. plantarum Inhibited the Expression of iNOS, COX2, and NF-kB Pathway Proteins

**Figure 3:**
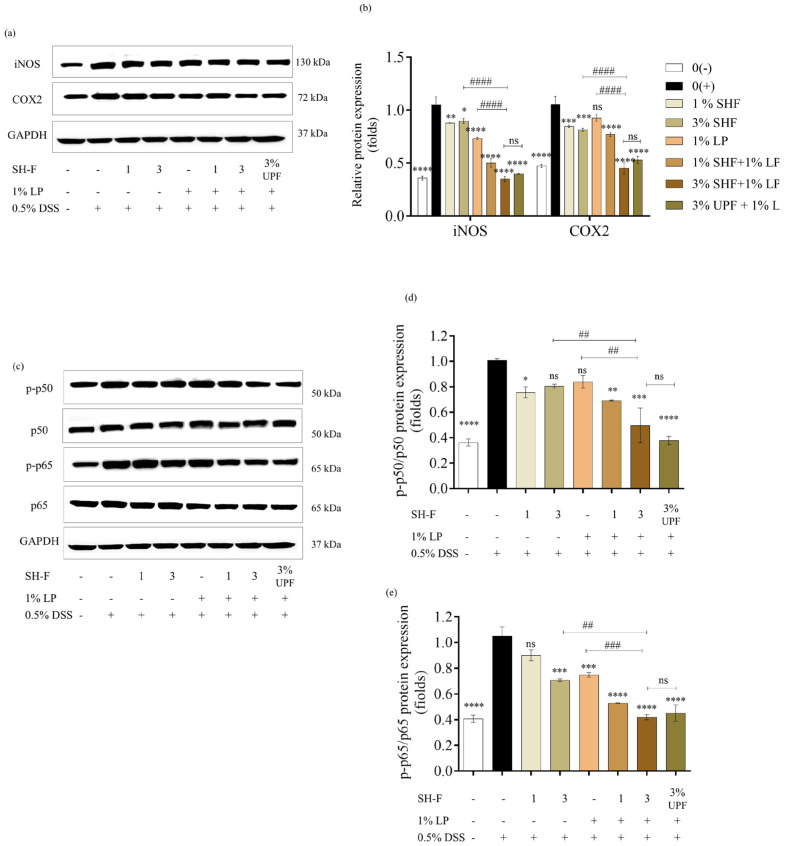
Effect of synbiotic treatment of SH-F and *L. plantarum* on iNOS, COX2, and NF-kB pathway protein expressions in 0.5% DSS-induced adult zebrafish. (**a**) Protein expressions of iNOS and COX2, (**b**) quantification iNOS, COX2, (**c**) protein expressions of NF-kB proteins, (**d**) quantification of p-p50, and (**e**) quantification of p-p65. The results are represented as means ± SD. Values are significantly different from the control group (0(+)) at * *p* < 0.05, ** *p* < 0.01, *** *p* < 0.005, and **** *p* < 0.0001, and from 3% SH-F+LP group at ## *p* < 0.01, ### *p* < 0.005, and #### *p* < 0.0001. (SH-F: *S. horneri* fucoidan, LP: *L. plantarum*, UPF: *U. pinnatifida* fucoidan).

Western blot results revealed an upregulation of iNOS and COX2 expression in zebrafish exposed to 0.5% DSS compared to the untreated group. The experimental diet containing SH-F and *L. plantarum* downregulated the expression levels of iNOS and COX2 (Figure 3a,b).

Expression of proteins involved in the NF-κB signaling pathway was measured to clarify the mechanism of the anti-inflammatory effect of the synbiotic treatment of SH-F and *L. plantarum*. According to the results, 0.5% DSS stimulation significantly upregulated the TLR4 expression and the phosphorylation of p65 and p50. However, the synbiotic treatment of SH-F and *L. plantarum* resulted in downregulated TLR4, p-p65, and p-p50 expressions in the intestine. The synbiotic treatment of SH-F and *L. plantarum* showed superior activity compared to their individual treatments (Figure 3c–e).

### 2.9. Synbiotic Treatment of SH-F and L. plantarum Restored Intestinal Barrier Function

**Figure 4:**
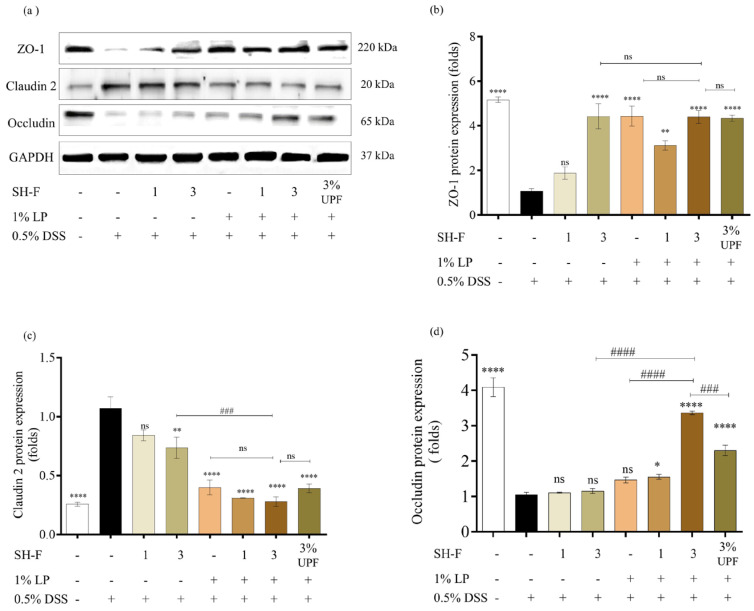
Synbiotic effect of SH-F and *L. plantarum* on DSS-induced alterations in tight junction proteins in 0.5% DSS-induced adult zebrafish. (**a**) Protein expressions of tight junction proteins, (**b**) quantification ZO-1, (**c**) quantification of claudin-2, and (**d**) quantification of occludin. The results are represented as means ± SD. Values are significantly different from the control group (0(+)) at * *p* < 0.05, ** *p* < 0.01, and **** *p* < 0.0001, and from 3% SH-F+LP group at ### *p* < 0.005, and #### *p* < 0.0001. (SH-F: *S. horneri* fucoidan, LP: *L. plantarum*, UPF: *U. pinnatifida* fucoidan).

Intestinal barrier function was evaluated based on the expression levels of tight junction proteins (TJ proteins). The 0.5% DSS exposure led to significant alterations in TJ proteins expression in zebrafish (Figure 4). The expression of key TJ proteins, including ZO-1 and occludin, exhibited a substantial reduction as a result of the 0.5% DSS-exposed group compared to the untreated group. Conversely, the protein expression of claudin-2 showed an increase in response to 0.5% DSS exposure. However, the synbiotic treatment demonstrated improved occluding and ZO-1 expression while downregulating claudin-2 expression.

### 2.10. SH-F and L. plantarum Synbiotic Treatment Inhibited the Gene Expression of Pro-Inflammatory Cytokines in DSS-Induced Zebrafish UC Model

**Figure 5:**
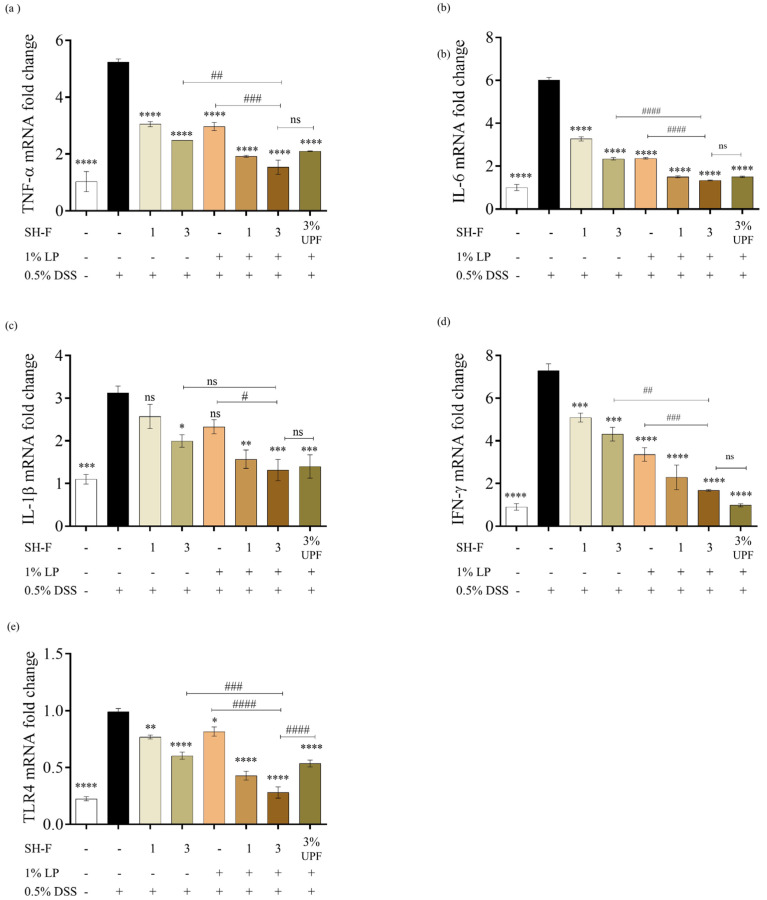
Synbiotic effect of SH-F and *L. plantarum* on the relative pro-inflammatory gene expression and TLR4 expression in 0.5% DSS-induced zebrafish. (**a**) TNF-ɑ mRNA expression, (**b**) IL-6 mRNA expression, (**c**) IL-1β mRNA expression, (**d**) IFNɤ mRNA expression, and (**e**) TLR4 mRNA expression. The results are represented as means ± SD. Values are significantly different from the control group (0(+)) at * *p* < 0.05, ** *p* < 0.01, *** *p* < 0.005, and **** *p* < 0.0001, and from 3% SH-F+LP group at # *p* < 0.05, ## *p* < 0.01, ### *p* < 0.005, and #### *p* < 0.0001. (SH-F: *S. horneri* fucoidan, LP: *L. plantarum*, UPF: *U. pinnatifida* fucoidan).

The gene expression analysis of zebrafish adult intestines subjected to 0.5% DSS exposure revealed significant alterations in the gene expressions linked to inflammatory responses and inflammatory signaling transduction including IL-1β, IL-6, TNF-α, IFNɤ, and TLR4 (Figure 5). Notably, the 0.5% DSS-exposed group resulted in increased expression levels of these genes in zebrafish intestines. The individual treatments with SH-F or *L. plantarum* resulted in a noteworthy reduction in their expression; however, the synbiotic treatment groups showed the highest inhibition of these pro-inflammatory gene expressions compared to their individual treatments.

### 2.11. SH-F and L. plantarum Combined Treatment Regulated Microbial Composition and Prevented Microflora Dysbiosis

**Figure 6:**
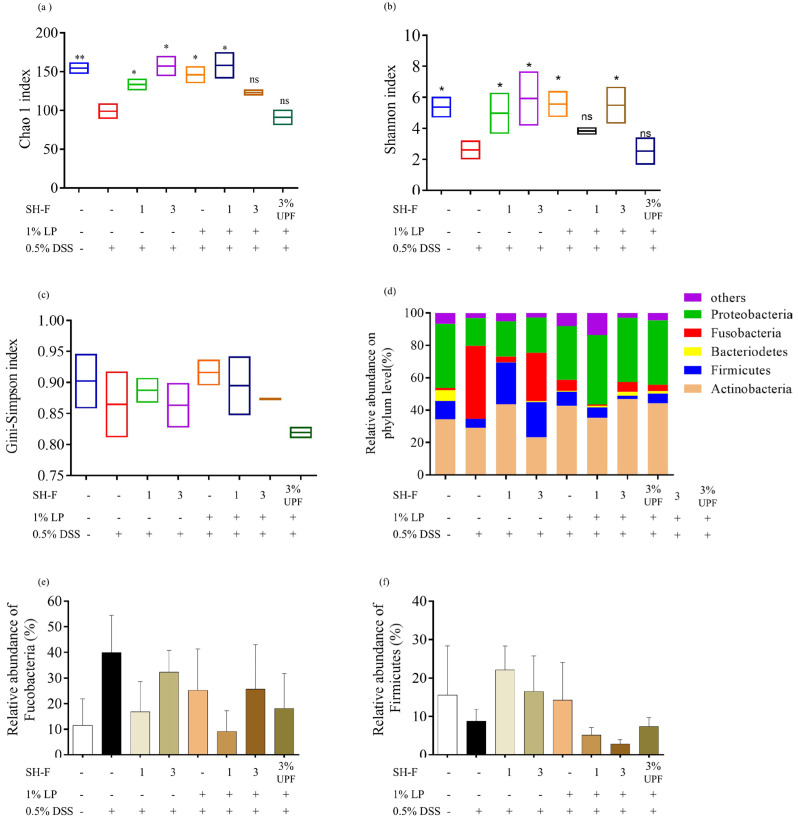
Synbiotic effect of SH-F and *L. plantarum* on the alpha diversity gut microbiome composition of 0.5% DSS-induced zebrafish. (**a**) Chao1 index, (**b**) Shannon index, (**c**) Gini Simpson index, (**d**) phylum-level analysis of microbiome composition, (**e**) relative abundance of *Fusobacteria*, and (**f**) relative abundance of *Firmicutes*. The results are represented as means ± SD. Values are significantly different from the control group (0(+)) at * *p* < 0.05, ** *p* < 0.01(SH-F: *S. horneri* fucoidan, LP: *L. plantarum*, UPF: *U. pinnatifida* fucoidan).

The dysbiosis of gut microbiota is a recognized factor contributing to the pathogenesis of IBD. The zebrafish intestines were subjected to 16S rRNA sequencing, and the changes in gut microbiota abundance were evaluated based on alpha diversity using the Chao1 (Figure 6a), Shannon (Figure 6b), and Simpson (Figure 6c) indices. The 0.5% DSS-exposed group showed lower values of these indices compared to the untreated group. The treatment of SH-F, *L. Plantarum*, and their combined treatment resulted in higher values compared to the 0.5% DSS-stimulated group.

The modification of the microbial community was quantified at the phylum level by 16S rRNA gene sequencing (Figure 6d). The groups mainly consisted of *Actinobacteria* and *Proteobacteria*, *Fusobacteria*, *Firmicutes*, and *Bacteroidetes* which comprised more than 90% of the total bacterial community. Compared to the untreated group, the abundance of Bacteroidetes decreased markedly after 0.5% DSS treatment, and the enhancing effect of synbiotic treatment was observed with increasing *Bacteroidetes* abundance in sample-treated groups. In contrast, the 0.5% DSS-treated group showed a higher abundance of Fusobacteria. However, synbiotic treatment resulted in reduced *Fucosabacteria* abundance in zebrafish (Figure 6e). The effect of 0.5% DSS stimulation was strong on the *Firmicutes* abundance in the zebrafish gut. Interestingly, their abundance was noticeably restored when fed with individual treatments rather than the combined treatment of SH-F and *L. plantarum* (Figure 6f).

At the genus level, the 0.5% DSS exposure resulted in a significant difference in microbial composition in the zebrafish (Supplementary Figure S2). A total of 16 common species in all treated groups of zebrafish were selected to find the potential genera responsible for the effect of the *L. plantarum* and SH-F treatment. The 0.5% DSS exposure caused a significant reduction in commonly abundant genera and increased the abundance of *Aeromonas* and *Cetobacterium*. The synbiotic treatment induced greater recovery of diminished bacteria with increased abundance in *Lactobacillus*, *Plesiomonas*, *Sphingomonas*, and *Faecalibaculum*.

## 3. Discussion

Ulcerative colitis (UC) is a complex condition influenced by a combination of genetic and environmental factors. Therefore, the development of novel therapeutic approaches has become important to alleviate the symptoms and mortality associated with UC. UC is linked to three key factors as follows: (i) imbalances in gut microbiota, (ii) increased intestinal permeability, and (iii) dysregulated immune responses. The restoration of these disrupted functions within the digestive system emerges as a promising mechanism for future treatment [19]. In this context, synbiotics containing both probiotics and prebiotics have emerged as a novel therapeutic strategy for the prevention of intestinal dysbiosis and intestinal barrier disruption associated with mucosal inflammation [20]. Subsequently, probiotic supplements, specifically those belonging to the *Bifidobacterium* and *Lactobacillus* genera, have shown successful application in patients with UC [13]. We hypothesized that the synbiotic action of the *L. plantarum* and *S. horneri* fucoidan combination is more effective than their individual treatments for the prevention and/or treatment of UC.

We utilized Celluclast-assisted extraction to isolate sulfated polysaccharides from *S. horneri*, followed by purification of fucoidan using DEAE anion-exchange chromatography. The sixth fucoidan fraction exhibited higher polysaccharide and sulfate contents. Notably, the progressive decrease in protein and polyphenol content across successive fractions highlights the effectiveness of the anion exchange purification process [21]. Biological activities exhibited by fucoidan mainly depend on the presence of sulfate groups and their location [22]. Therefore, the higher sulfate content and fucose content in SH-F are associated with its enhanced biological activity. FTIR results revealed that the intensity of the sulfate substitution peak increased from unpurified SHC to purified SH-F. This finding aligns with the increased sulfate content in SH-F. Molecular weight analysis showed that our sample possesses a molecular weight of 254 kDa. It is proven that higher molecular weight fucoidan possesses improved biological activities such as anti-inflammatory, immunomodulatory, antioxidant, and prebiotic activities [23,24,25]. In the SH-F, the presence of monomers Fuc_1_(SO_3_)_1_, Fuc_2_(SO_3_)_1_, Gal_1_Fuc_1_(SO_3_)_1_ was identified, indicating structural units in which fucose and galactose are conjoined repetitively. Fucoidans are sulfated homo- and heteropolysaccharides whose structures vary greatly depending on the algal species, age, geographic location, and season of harvest [26]. The most widely accepted structural models describe fucoidans as having a linear or branched sulfated L-fucopyranoside backbone, typically linked by alternating α-(1→3) and α-(1→4) glycosidic bonds [27].

The synbiotic treatment of *L. plantarum* and *S. horneri* fucoidan did not cause a significant increase or decrease in body weight between fish fed with the synbiotic treatment and commercial feed. This indicates that the sample diet neither promoted nor inhibited growth under basal conditions. Moreover, no mortality was observed throughout the synbiotic feeding as well as during DSS exposure. This shows that the synbiotic treatment was well tolerated and did not adversely affect their survival. These observations suggest that the synbiotic formulation is safe and does not cause any deleterious effects on growth or viability in zebrafish.

Moreover, the synbiotic treatment effectively ameliorated the intestinal inflammation in our adult zebrafish model. The results showed that DSS treatment induced a significant reduction in gut length, representing the negative effects of DSS-induced inflammation on intestinal morphology when compared to the control group. The intestinal barrier is the primary site for interacting with the external environment, mainly food and microbes, which plays a fundamental role in health and disease. The normal intestinal mucosal barrier is composed of mechanical, chemical, immune, and biological barriers. However, during UC, the function of the intestinal barrier is compromised. The term “chemical barrier” refers to the protective mucin layer that coats the surface of intestinal epithelial cells. This layer primarily consists of Mucin-2, a mucin protein secreted by goblet cells, along with digestive secretions and bacteriostatic substances produced by beneficial bacteria residing in the intestinal lumen [28]. This plays an important role in separating the internal and external environments of the intestinal tract, providing protection from infiltration of harmful substances from the intestinal lumen. Previous studies showed that 0.5% DSS exposure resulted in decreased goblet cell number and increased tight junction permeability [29]. These results were consistent with our current findings. Histological examination with Alcian blue staining revealed a reduction in the number of goblet cells in the 0.5% DSS group due to tissue damage. Goblet cells are instrumental in producing mucus, which forms a protective barrier for the intestinal lining [30]. A decrease in goblet cells is concerning because it indicates a compromised mucosal defense in the gut, leaving it vulnerable to further damage. Conversely, the number of goblet cells increased following the supplementation of the synbiotic diet. Sun et al. (2020) made a similar observation, highlighting that *L. plantarum* has the capacity to enhance the intestinal mucosal chemical barrier by elevating the levels of mucin-2 [31].

The TJ proteins maintain the intestinal mucosal cell barrier function and regulate the entry of detrimental substances, including bacteria and endotoxins, into the bloodstream through the intestinal mucosa [32]. Notably, the disruption of the mechanical barrier in patients with IBD can be attributed to abnormal expression of TJ proteins [33]. This includes the reduction in essential proteins like ZO-1 and occludin and an increase in claudin-2. Given the significant improvement in histopathology achieved by the synbiotic treatment of *L. plantarum* and *S. horneri* fucoidan, we analyzed the regulatory role of synbiotic treatment on TJ protein expression using Western blot analysis. We observed significant downregulation of occludin and ZO-1 and upregulation of claudin-2 expression level in our zebrafish model, consistent with previous reports [34,35].

UC is a chronic inflammatory condition, and its pathological process involves several inflammatory cells that produce inflammatory cytokines such as TNF-α, IL-1β, and IL-6, which are associated with chronic inflammation and tissue damage [36]. TNF-α is responsible for many immune and inflammatory responses, and it initiates inflammation by altering TJ structure and disrupting epithelial barrier function [37]. Moreover, IL-1β is responsible for the triggering of pro-inflammatory mediators like chemokines and cytokines, which results in extensive inflammatory responses. It is reported that colon tissues of UC patients express higher levels of IL-1β mRNA [38]. The levels of pro-inflammatory cytokines in our 0.5% DSS-exposed adult zebrafish gut were significantly higher than the untreated group, indicating progression of inflammation. These cytokine expressions were strongly reduced by the synbiotic treatment compared to their individual treatments.

In the DSS-induced UC model, the intestinal barrier is disrupted by DSS, and the intestinal microbes enter the immune system, which triggers a series of inflammatory reactions. This is a result of the stimulation of Toll-like receptor 4 (TLR4) by its ligands and leads to the activation of the NF-kB signaling pathway [39]. Normally, the TLR4 expression level is low in normal tissues and upregulated during inflammation [39]. In our study, exposing adult zebrafish to 0.5% DSS resulted in upregulation of TLR4 expression in intestinal tissues, while its expression was the lowest in the group treated with a synbiotic diet. These results align with findings from a study conducted on *Clostridium butyricum* and chito-oligosaccharides synbiotic-treated DSS-induced UV mouse model [40].

The pathogenesis of IBD/UC is correlated with the dysbiosis of the gut microbiome in patients. It is reported that IBD patients show an altered gut microbiota composition compared to healthy individuals with lower microbial diversity [41]. Numerous studies have proven that probiotics improve the clinical outcomes of UC patients through influencing host gut microbiota [42]. Healthy individuals contain a higher proportion of *Bacteroidota* and *Firmicutes* phyla and a smaller proportion of *Proteobacteria* and *Fusobacteria* [43]. Our present study showed that exposure to 0.5% DSS significantly reduced α diversity, leading to a reduction in microbiome diversity and richness. Moreover, it also resulted in a higher difference in the structure of gut microbiota between the treatment groups. In particular, the synbiotic treatment of *L. plantarum* and *S. horneri* fucoidan remarkably increased the gut microbiota diversity and reduced gut microbiota structural differences in gut microbiota. There was an increase in the abundance of potentially beneficial probiotic species in the genus *Bacteroides* and *Lactobacillus*, some of which are known to reduce inflammation or are associated with various health benefits [44]. These bacteria produce antimicrobial substances, compete with pathogens for epithelial and mucin-binding sites, and have been shown to attenuate symptoms and maintain remission of UC [45]. In addition, it was found that DSS exposure in zebrafish results in increased abundance of *Aeromonas* and *Staphylococcus*, which are closely related to the development of colitis [46]. In future studies, follow-up experiments will be conducted with a larger sample size (*n* ≥ 5), and other mammalian or intestinal organoid models will be employed to further validate these findings. To directly evaluate the causal role of microbiota in mediating the barrier-protective effects of SH-F, fecal microbiota transplantation (FMT) or antibiotic-treated zebrafish models are required and will be used for future studies. These efforts will enhance statistical robustness and provide a more comprehensive understanding of the protective effects of SH-F and *L. plantarum* on the intestinal barrier and microbiota.

In summary, these findings emphasize the interplay between gut microbiota, the gut barrier, and immune system, and how these factors collectively contribute to the development and management of UC. Our study demonstrates that the synbiotic treatment of *S. horneri* fucoidan and *L. plantarum* contributes to the inhibition of UC-related negative effects in the gut by modulating the gut microbiome, reducing inflammation, and potentially influencing host health.

## 4. Materials and Methods

### 4.1. Chemicals and Reagents

All the organic solvents (HPLC grade) used in the experiments were purchased from Sigma-Aldrich (St Louis, MO, USA). Probiotic bacterium Lactobacillus plantarum (KCCM 12116) was purchased from the Korean Culture Center for Microorganisms (Jeongeup-si, Jeollabuk-do, South Korea). Primary and secondary antibodies were purchased from Cell Signalling Technology (Boston, MA, USA) and Santa Cruz Biotechnology (Dallas, TX, USA). Enhanced chemiluminescence reagents were obtained from Amersham (Amersham, Wilmington, USA). *Undaria pinnatifida* fucoidan was purchased from Sigma-Aldrich (St Louis, MO, USA). Diethylaminoethyl cellulose (DEAE-cellulose, SepharoseTM Fast Flow) was purchased from Cytiva (Marlborough, MA, USA) to separate polysaccharides. All other chemicals and solvents used in this study were of analytical grade.

### 4.2. Preparation of S. horneri Fucoidan

*S. horneri* fucoidan was prepared, as described in our previous study [17]. Seaweed was collected and identified by morphological characteristics according to standard taxonomic keys for brown algae. Collected seaweed was washed, dried, and ground. The samples were then depigmented using 95% ethanol, followed by Celluclast-assisted enzymatic extraction at pH 4.5–5.0 and 50 °C for 24 h. Alginates were removed from the extract using CaCl2 addition, and sulfated polysaccharides (SHCP) were precipitated with 95% ethanol at 4 °C. For fucoidan purification, SHCP was dissolved in a sodium acetate buffer and applied to a DEAE-cellulose column. Elution was carried out with NaCl in a stepwise gradient (0.2 M to 2.0 M), with fractions collected, analyzed, and pooled into six groups, then lyophilized. Finally, dialysis was performed to remove ionic contaminants, with repetition until the conductivity was comparable to distilled water [16].

### 4.3. S. horneri Fucoidan on L. plantarum Growth

For the selection of the fucoidan fraction showing the best prebiotic activity, *S. horneri* fucoidan was treated with probiotic bacteria to measure bacterial growth. The probiotic bacterial strain, *L. plantarum* (KCCM 12116), was cultured in De Man–Rogosa–Sharpe (MRS) broth and incubated at 37 °C. The extracted S. *horneri* fucoidans were screened with *L. plantarum* for their growth-enhancing or inhibiting activity using the colony-counting assay [47]. *L. plantarum* was cultured in MRS broth at 37 °C overnight, and the density of the cells was adjusted to 10^5^ colony-forming units/mL (CFU/mL). An equal number of bacterial suspensions (100 µL) were added to the MRS culturing with 200 µg/mL fucoidan fractions and incubated overnight at 37 °C in an incubator. The probiotic cultured in MRS without fucoidan was used as a control. After the incubation period, bacterial suspension was diluted and incubated on MRS agar plates. The number of colony-forming units (CFU) was counted, and growth rates were calculated. The fraction showing the best growth-enhancing activity was selected for subsequent studies.

### 4.4. Chemical Composition and Monosaccharide Composition Analysis of S. horneri Fucoidan

Polysaccharide, protein, sulfate, and polyphenol of the fucoidan fractions were measured using the Standard Analysis Community Association (AOAC) procedures. Total polysaccharide content was measured using the phenol-sulfuric method, and protein content was measured using the Lowry assay. The BaCl_2_ method was used to measure the sulfate content, and the Folin–Ciocalteu method was used in polyphenol analysis [48]. The monosaccharide composition of the *S. horneri* fucoidan fraction, hydrolyzed using trifluoroacetic acid (TFA), was analyzed using RP-HPLC analysis [49].

### 4.5. Fourier Transform Infrared Spectroscopy (FTIR) Analysis and Molecular Weight Determination

SHC, SHCP, and SH-F were assessed using an FTIR spectrophotometer from Thermo Scientific NicoletTM 6700, MA, USA. Fucoidan samples were milled into powder and mixed with spectroscopic grade KBr powder and compressed into a pellet for FTIR analysis. Scanning was performed with a resolution of 4 cm^−1^, between 500 and 3500 cm^−1^. The spectra were compared to the commercial fucoidan that was being researched concurrently [50].

The molecular weight of fucoidan was measured by size-exclusion chromatography (SEC) using an HPLC system fitted with online multi-angle static light scattering (MALS) and viscometry (VISC) detectors [51]. The measurements were performed at ambient temperature using PL aquagel-OH MIXED-H column. The column outlet was connected to a Wyatt Dawn HELEOS-II (18 Angles) multi-angle laser light scattering photometer (Wyatt, USA) (λ0 = 663.8 nm), followed by an Optilab T-rEX differential refractometer. The eluent was 0.15 M NaNO_3_/0.01 M EDTA (pH = 6, I = 0.17 M), and the flow rate was 0.5 mL/min. Samples were filtered (pore size = 0.8 μm) before injection. The injection volume was 100 μL, and the sample concentration was adjusted to obtain the best possible light-scattering signal without influencing the RI profile (overloading). The data were collected and processed using the Astra (v. 6.1) software (Wyatt, CA, USA).

### 4.6. Methylation Analysis

Methylation analysis was carried out according to a previously reported method with minor modifications [52]. *S. horneri* fucoidan was subjected to carboxyl reduction with NaBH_4_. The sample was then methylated with NaOH-DMSO and methyl iodide. The reaction mixture was first extracted with chloroform and then hydrolyzed with trifluoroacetic acid. After dissolving the hydrolysate in 1% (*w*/*w*) NaOH, the sample was then acetylated with acetic anhydride and pyridine. The acetylated products were extracted with chloroform and analyzed using a gas chromatograph–mass spectrometer (GC-MS) (Perkin–Elmer GC Clarus 500 system) using a DB-5MS column (30 m × 0.25 mm × 0.25 µm, Agilent (CA, USA)). The temperature program was as follows: held at 160 °C for 1 min, then increased from 160 °C to 250 °C at a rate of 8 °C/min and held for 3 min. The injector temperature was 280 °C, detector temperature 280 °C, and the carrier gas helium, at 1 mL/min. The ion source temperature was 280 °C, at the range of 30–500 *m*/*z*. GC–MS data acquisition and integration were performed using GC–MS Lab Solution (Version 4.30, Shimadzu, GCMS-QP201, Kyoto, Japan).

### 4.7. UPLC-Q-TOF MS/MS Analysis

The glycans of *S. horneri* fucoidan were analyzed by ultra-high performance liquid chromatography-quadrupole time-of-flight (UPLC-Q-TOF) MS/MS. The analyzed instrument used was Waters Xevo G2-XS Q-TOF (Waters, 34 Maple Street, Milford, MA, USA). The column was a Supel Carbon LC column (2.1 × 100 mm, 2.7 µm), and the mobile phase consisted of 10 mM ABC DW-10 mM ABC 90% acetonitrile (ACN) with a gradient method (flow rate 0.2 mL/min; 0 min 100:0 *v*/*v*; 0–5 min 100:0 *v*/*v*; 5–30 min 75:25 *v*/*v*; 30–35 min 50:50 *v*/*v*; 35–40 min 0:100 *v*/*v*; 40–45 min 0:100 *v*/*v*; 45–45.1 min 100:0 *v*/*v*; 45.1–50 min 100:0 *v*/*v*). Fucoidan mass analysis was performed in a negative mode in a Q-TOF MS/MS mass spectrometer coupled with a column specialized in glycan and oligosaccharide separation and showing high resolution. The condition of ESI-MS/MS was set to a scan range of 50 to 2000 *m*/*z* and a scan rate of 2 spectra/s, and helium was used at a gas temperature of 280 °C and 12 L/min as collision gas. The collision energy value was set to 6~90 V. MSe was selected as the scanning mode of mass spectrometry, and the exact *m*/*z* (decimal = 4) precursor ion values for all glycans were entered in the inclusion list of MSe modes, respectively. MS chromatogram and MS/MS spectrum were obtained through MassLynx v4.1 SCN888 (Waters Corporation, Wilmslow, UK) and data analysis was performed using UNIFI™ v1.8.0 (Waters Corp) [48].

### 4.8. Maintenance of Adult Zebrafish

The zebrafish experiment was approved by the Animal Care and Use Committee of Jeju National University (Approval No. 2023-0005).

Zebrafish were purchased from Seoul Aquarium, Korea. Fish were separated into tanks (260 mm × 155 mm × 160 mm and volume of 3.5 L) (15 fish per tank) under control conditions (29 °C with 14/10 h light and dark cycle) and acclimated for two weeks with temperature 28.5 ± 1 °C, salinity 0 g/L, and a flow rate of 700 mL/min. They were fed twice daily with tetrabits flakes (Tetrabits GmbH D-49324 Melle, Germany) with a feeding rate of 3% of body weight in feed per day.

### 4.9. Experimental Diet Preparation

The formulated commercial fish feed (Tetrabit Gmbh D49304, 47.5% crude protein, 6.5% crude fat, 2.0% crude fiber, 10.5% crude ash) was mixed with 1% pure culture of *L. plantarum* (*w*/*w*) and *S. horneri* fucoidan (1%, and 3%, (*w*/*w*)). Briefly, *L. plantarum* was cultured in MRS medium for 24 h, and cells were collected by centrifugation. Cells were washed with PBD twice and freeze-dried to obtain *L. plantarum* powder. Freeze-dried probiotic samples and freeze-dried SH-F were dissolved in autoclaved distilled water and mixed with commercial feed. The extruded fish feed was freeze-dried and made into a powder.

### 4.10. Zebrafish Experiment Design

Size- and age-matched zebrafish were separated into 8 groups as untreated (0(−)), 0.5% DSS (0(+)), 1% SH-F, 3% SH-F, 1% LP, 1% SH-F+1% LP, 3% SH-F + 1% LP, and 3% UPF +1% LP, respectively (15 fish/tank). Fish were fed with the diet twice a day for 14 days and stimulated with a 0.5% DSS bath for 8 h/day for 5 days to induce intestinal injury. Fish were then euthanized after fasting for 12 h. Intestines were dissected, and the length of the intestine was measured and used for subsequent analysis. During the experiment, the mortality of fish was recorded. A schematic diagram of the experimental design is given in Supplementary Figure S1.

### 4.11. Measurement of Body Weight

The body weight of zebrafish before and after sample diet feeding was recorded. Growth performance was analyzed using weight gain following the method given in a previous study [53].

### 4.12. Histological Analysis

Zebrafish intestines were subjected to H&E staining and Alcian blue staining. Briefly, the intestines were fixed in 10% formalin and dehydrated using alcohol to remove water from the tissues. The tissues were then cleared using xylene and impregnated with paraffin wax. Sections were deparaffinized using xylene and rehydrated using ethanol and distilled water. H&E staining and Alcian blue were used to stain the slides. Stained slides were dehydrated, mounted with coverslips, and examined with a microscope under a magnification of 40 to 1000× (Olympus, Tokyo, Japan) [16].

### 4.13. qPCR Analysis

The total RNA from zebrafish adult intestine in each group was extracted using a Tri-Reagent™ extraction kit (Sigma Aldrich, St. Louis, MO, USA). The RNA samples were then transcribed into cDNA using a cDNA Reverse Transcription Kit (Takara, Shiga, Japan). RT-qPCR amplification of cDNA was performed using a Thermal Cycler Dice Real-Time System (Takara, Japan) under the following conditions: enzyme activation at 95 °C for 10 s, followed by 40 cycles of denaturation at 95 °C for 5 s, and annealing at 58 °C for 10 s. The reaction was carried out in a 10 μL volume containing 3 μL of cDNA, 5 μL of the 2× TaKaRa ExTaq SYBR Premix (TaKaRa, BIO INC, Japan), 0.4 μL of primers, and 12 μL of RNase/DNase-free water [54]. The primers used in this study are provided in Supplementary Table S1.

### 4.14. Western Blot Analysis

Intestines of experimental zebrafish were extracted, and protein lysates were prepared. Protein concentrations were measured using the BCA protein assay kit. Tight junction proteins, cyclooxygenase-2 (COX2), inducible nitric oxide synthase (iNOS), and NF-κB pathway protein expression levels were analyzed by the Western blot technique [55]. Extracted proteins were subjected to sodium dodecyl sulfate–polyacrylamide gel electrophoresis, and protein bands were transferred to nitrocellulose membranes (Bio-Rad, Hercules, CA, USA). Membranes were cut horizontally based on molecular weight markers and each section was blocked with 5% skimmed milk for 2 h and incubated with primary and secondary antibodies. Enhanced chemiluminescence reagents (Cyanagen Srl, Bologna, Italy) were used to develop the blots, and a FUSION SOLO Vilber Lourment system (Paris, France) was used to photograph the developed blots. ImageJ software v1. 54d was used to aid in quantifying band intensities [56].

### 4.15. 16S rRNA Sequencing and Data Analysis

Gut microbiota in zebrafish was analyzed. Total genomic DNA from zebrafish intestinal contents was extracted using a commercial kit (QIAGEN, Hilden, Germany), according to the manufacturer’s instructions. PCR amplification was performed with the extracted DNA using fusion primers targeting from the V3 to the V4 regions of the 16S rRNA gene. The amplification, sequencing, and library preparation workflow was conducted using Herculase II fusion DNA polymerase Nextera XT Index Kit V2, following the 16S metagenomic sequencing library preparation Part # 15044223 Rev. B protocol in the Illumina^®^ MiSeq^®^ platform at Macrogen (Seoul, South Korea), yielding paired-end reads [34].

The EzBioCloud 16S rRNA database [57] was used for taxonomic assignment using the usearch_global command of VSEARCH [58] followed by more precise pairwise alignment. Chimeric reads were filtered on reads with <97% similarity by reference-based chimeric detection using the UCHIME algorithm [59] and the non-chimeric 16S rRNA database from EzBioCloud. After chimeric filtering, reads that were not identified to the species level (with <97% similarity) in the EzBioCloud database were compiled and the cluster fast command was used to perform de novo clustering to generate additional OTUs [58]. Finally, OTUs with single reads (singletons) were omitted from further analysis. The secondary analysis, which included diversity calculation and biomarker discovery, was conducted by an in-house program named CJ Bioscience, Inc. (Seoul, Republic of Korea). Relative abundance was calculated to identify variations in microbial community structure. Microbial diversity was evaluated using alpha diversity metrics, including the Chao-1 richness estimator, Shannon diversity index, and Simpson index, as well as beta diversity analyses to assess differences in community composition across samples [60,61].

### 4.16. Statistical Analysis

Statistical analysis was performed using one-way ANOVA followed by Dunnett’s multiple comparisons test with GraphPad Prism 10 (GraphPad Software, Inc., San Diego, CA, USA). Unless otherwise specified, all experiments were conducted in triplicate, and data are presented as the mean ± standard error of the mean (SEM). For 16S rRNA sequencing analysis, two biological replicates per group were used for preliminary exploratory purposes. Differences were considered statistically significant when *p* < 0.05.

## 5. Conclusions

UC is a complex condition influenced by various factors, and its effective treatment remains a challenge. The study explored a novel approach to address UC by focusing on the restoration of disrupted functions within the digestive system, including the gut microbiota, intestinal barrier integrity, and immune responses. Probiotics and prebiotics were investigated as potential therapeutic agents. Using an adult zebrafish model, the study revealed that intestinal length in adult zebrafish exposed to DSS treatment demonstrated the severity of gut inflammation, but synbiotic treatment with SH-F and *L. plantarum* restored intestinal length, highlighting the treatment’s efficacy. The study also showed that this combined treatment improved the mechanical barrier of the intestinal mucosal barrier, involving TJ proteins. The treatment effectively inhibited the NF-κB pathway and regulated pro-inflammatory cytokines. Synbiotic treatment with SH-F and *L. plantarum* improved the diversity and composition of gut microbiota, which play a crucial role in inflammation and intestinal health. In conclusion, the study demonstrated that synbiotic treatment with SH-F and *L. plantarum* has the potential to restore the microbial barrier, reduce inflammation, and influence overall host health, offering promise for protecting the intestinal barrier and managing UC.

## Figures and Tables

**Table 1:** Glycosidic linkage composition of SH-F.

	Retention Time (min)	Partially Methylated Alditol Acetates (PMAA)	Methylated Sugar	Mass Fragment (*m*/*z*)	Molar Ratio	Type of Linkage
1	3.95	1,5,6-Tri-O-acetyl-1-deuterio-2,3,4-tri-O-methyl-D-galactitol	6-linked-D-galactopyranosyl residue	41, 43, 85, 103, 131, 160, 176, 198, 220, 258, 272, 298	0.11	→6)-Gal(1→
2	4.11	1,4,5-Tri-O-acetyl-1-deuterio-6-deoxy-2,3-di-O-methyl-L-galactitol	4-linked-6-deoxy-L-galactopyranosyl (Fuc) residue	41, 43, 85, 103, 120, 145, 176, 198, 220, 258, 268, 300	0.16	→4)-Fucp(1→
3	4.25	1,3,5-Tri-O-acetyl-1-deuterio-6-deoxy-2,4-di-O-methyl-L-galactitol	3-linked-6-deoxy-L-galactopyranosyl(Fuc) residue	41, 43, 85, 103, 119, 148, 190, 213, 220, 258, 272, 300	0.44	→3)-Fucp(1→
4	5.33	1,4,5,6-Tetra-O-acetyl-1-deuterio-2,3-di-O-methyl-D-galactitol	4,6-linked-D-galactopyranosyl residue	41, 43, 72, 114, 118, 161, 185, 199, 241, 243, 288, 326, 368	0.13	→4,6)-Gal(1→
5	9.25	1,3,4,5-Tetra-O-acetyl-1-deuterio-6-deoxy-2-O-methyl-L-galactitol	3,4-linked-6-deoxy-L-galactopyranosyl (Fuc) residue	41, 43, 73, 97, 129, 157, 185, 213, 227, 258, 281, 299	0.16	→3,4)-Fucp(1→
				Sum of molar ratio	1	

## Data Availability

The original data presented in the study are included in the article and the Supplementary Material; further inquiries can be directed to the corresponding author.

## Supplementary Materials

The following supporting information can be downloaded at: https://www.mdpi.com/article/10.3390/md23100372/s1, Figure S1: Schematic diagrams of adult zebrafish experimental designs; Figure S2: Growth of *L. plantarum* in *S. horneri* fucoidan-containing medium. The results are represented as means ± SD. Values are significantly different from the control group (0(+)) at **** *p* < 0.0001, and from frac 06 at #### *p* < 0.0001; Figure S3: The heatmap of relative abundances of core microbiota at the genus level in symbiotic diet-fed and 0.5% DSS-induced zebrafish. (SHF: *S. horneri* fucoidan, LP: *L. plantarum*, UPF: *U. pinnatifida* fucoidan); Table S1: Primers used in the current study; Table S2: Yield and purity of isolated fractions from DEAE chromatography; Table S3: Chemical and monosaccharide composition of *S. horneri* fucoidan.
