# Supplementary material for: The Synergistic Role of Sargassum horneri Fucoidan and Lactobacillus plantarum: Microbiome and Gut Barrier Restoration in Zebrafish Colitis
Source: Mar Drugs. 2025 Sep 25;23(10):372. doi: 10.3390/md23100372 (PMC12565283; doi:10.3390/md23100372)
Supplement: Supplementary file 1 [file marinedrugs-23-00372-s001.zip › marinedrugs-3884393-supplementary tables.pdf]

Supplementary Table S1. Primers used in the current study

Gene	Forward	Reverse
TNF-α	GCGCTTTTCTGAATCCTACG	TGCCCAGTCTGTCTCCTTCT
IL-1β	ATCAAACCCCAATCCACAGAG T	GGCACTGAAGACACCACGTT
IL-6	TCAACTTCTCCAGCGTGATG	TAAAGCACTCCACAACCCCA
TLR4	ACAGATCACCTGGACAGCAAG	TGCTTGAAAGTCCCGCATGT
**I**FN-γ	AGCTCTTCCTCATGGCTGTT	GGTCAACCAACCACAAGCAT

Supplementary Table S2. Yield and purity of isolated fractions from DEAE

Fraction	Yield (%)	Purity (%)
SHCP	38.63 ± 0.02	65.17
SH-F1	3.36 ± 1.32	84.52
SH-F2	2.37 ± 0.78	83.99
SH-F3	3.88 ± 3.00	88.24
SH-F4	2.66 ± 0.45	89.65
SH-F5	3.24 ± 0.49	94.34
SH-F6	3.95 ± 0.82	94.3

chromatography

Supplementary Table S3. Chemical and monosaccharide composition of *S. horneri* fucoidan

	Polysaccharides %	Protein %	Polyphenol %	Sulfate %	Monosaccharide composition (%)						
					Fucose	Mannose	Xylose	Glucose	Rhamnose	Galactose	Mannuronic
**S H C P**	50.17 ± 0.44	4.11±0.14	3.29 ± 0.22	15.00 ± 0.24	30.78 ± 0.11	7.83 ± 0.17	11.33 ± 0.07	13.28 ± 0.71	6.37± 0.11	21.93 ± 0.00	8.26 ± 0.35
**SHF**	62.09 ± 0.47	0.25 ± 0.10	0.85 ± 0.21	32.21 ± 0.12	49.43 ± 0.17	5.95 ± 0.10	8.77± 0.01	5.79 ± 0.14	7.51 ± 0.16	20.05 ± 0.31	nd
