# Supplementary material for: The Synergistic Role of Sargassum horneri Fucoidan and Lactobacillus plantarum: Microbiome and Gut Barrier Restoration in Zebrafish Colitis
Source: Mar Drugs. 2025 Sep 25;23(10):372. doi: 10.3390/md23100372 (PMC12565283; doi:10.3390/md23100372)
Supplement: Supplementary file 1 [file marinedrugs-23-00372-s001.zip › marinedrugs-3884393-supplementary figures.pptx]

## Slide 1
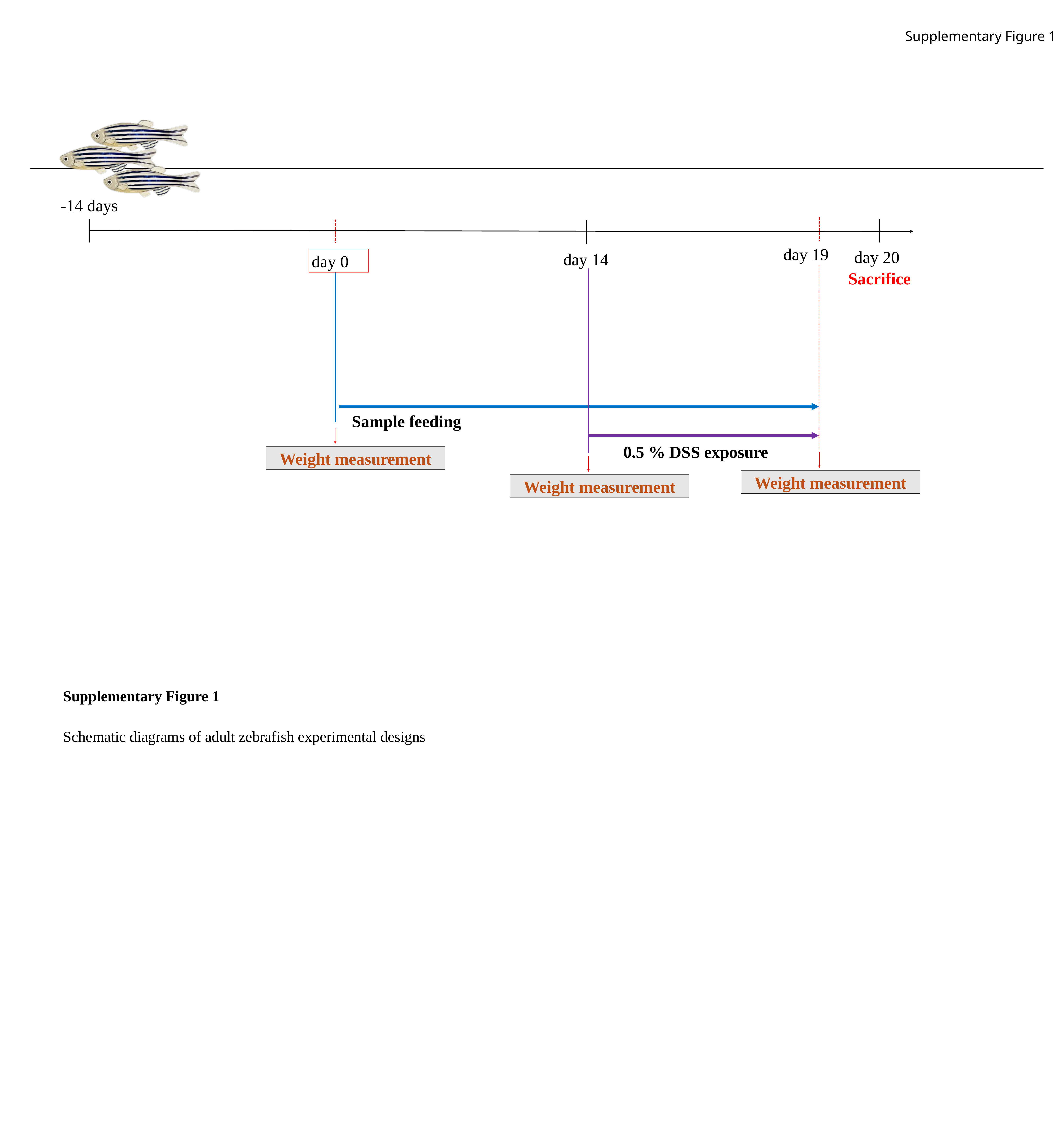

Supplementary Figure 1
-14 days
 day 19
day 14
day 0
Sample feeding
0.5 % DSS exposure
Weight measurement
 day 20
Sacrifice
Weight measurement
Weight measurement
Supplementary Figure 1
Schematic diagrams of adult zebrafish experimental designs

## Slide 2
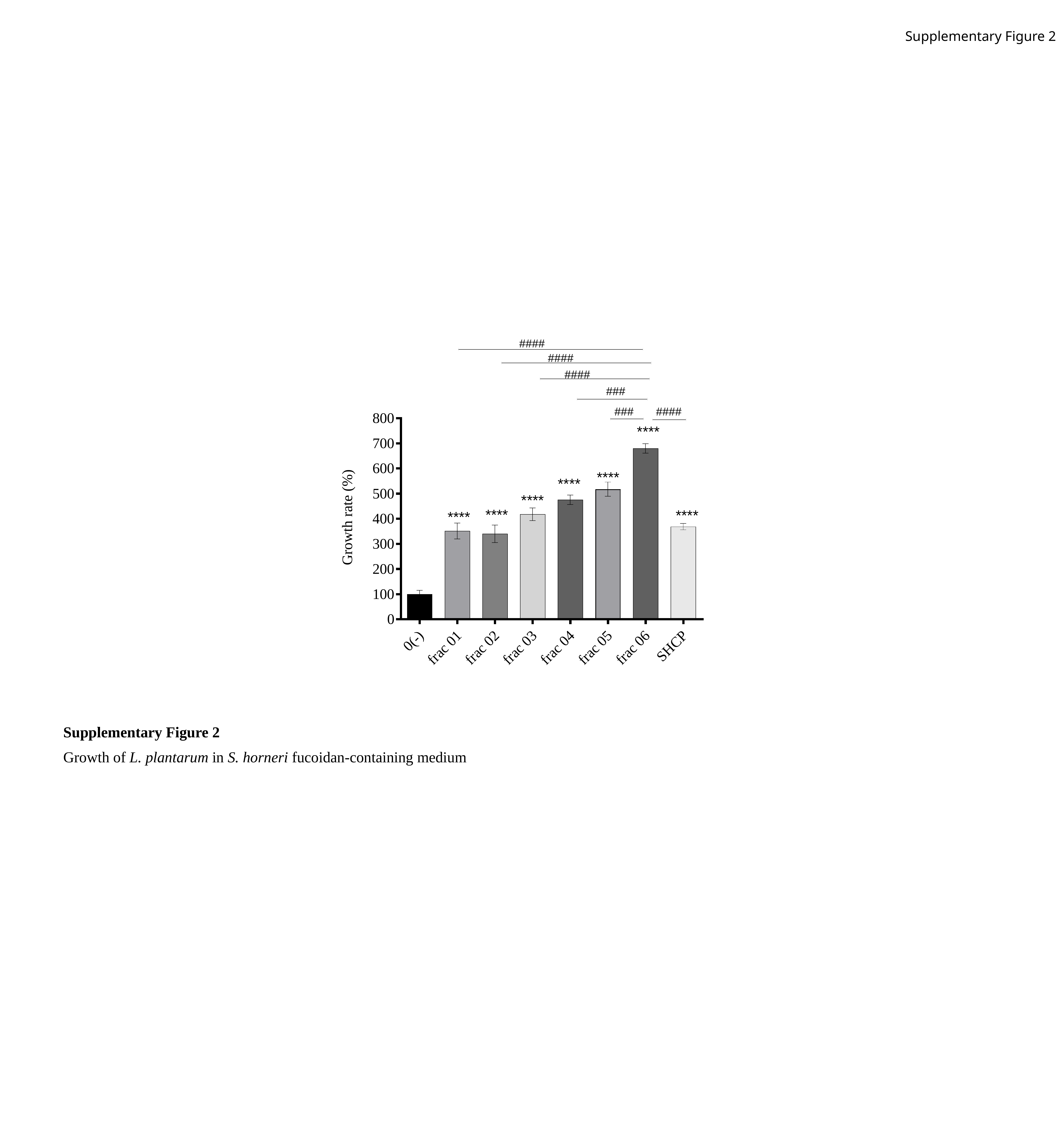

Supplementary Figure 2
Supplementary Figure 2
Growth of L. plantarum in S. horneri fucoidan-containing medium

## Slide 3
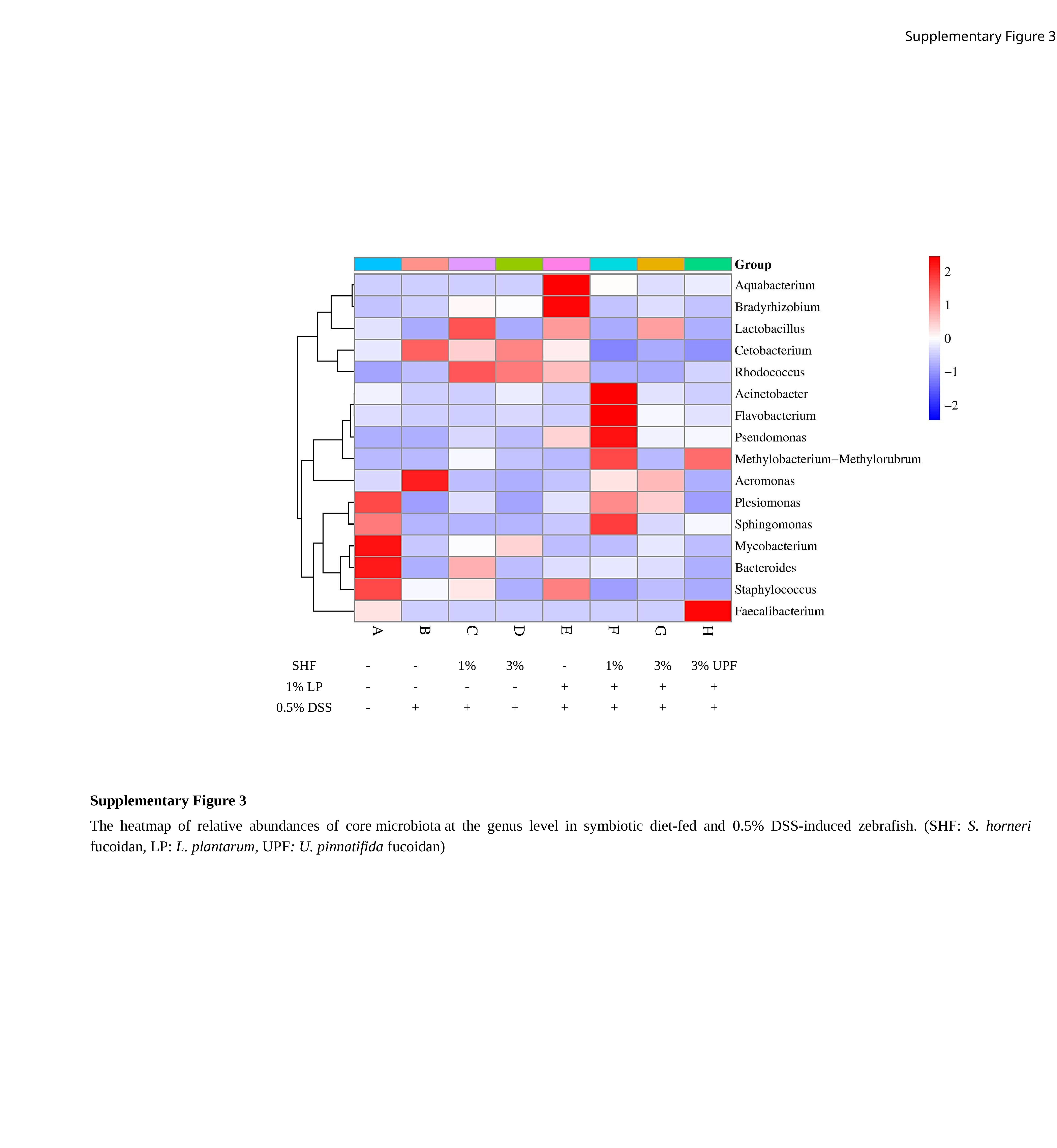

Supplementary Figure 3
| SHF | - | - | 1% | 3% | - | 1% | 3% | 3% UPF |
| --- | --- | --- | --- | --- | --- | --- | --- | --- |
| 1% LP | - | - | - | - | + | + | + | + |
| 0.5% DSS | - | + | + | + | + | + | + | + |
Supplementary Figure 3
The heatmap of relative abundances of core microbiota at the genus level in symbiotic diet-fed and 0.5% DSS-induced zebrafish. (SHF: S. horneri fucoidan, LP: L. plantarum, UPF: U. pinnatifida fucoidan)
